# GDF15 deficiency exacerbates chronic alcohol- and carbon tetrachloride-induced liver injury

**DOI:** 10.1038/s41598-017-17574-w

**Published:** 2017-12-08

**Authors:** Hyo Kyun Chung, Jung Tae Kim, Hyeon-Woo Kim, Minjoo Kwon, So Yeon Kim, Minho Shong, Koon Soon Kim, Hyon-Seung Yi

**Affiliations:** 1 Research Center for Endocrine and Metabolic Diseases, Chungnam National University School of Medicine, 282 Munhwaro, Daejeon, 35015 Republic of Korea; 2 Research Institute for Medical Sciences, Chungnam National University School of Medicine, 266 Munhwaro, Daejeon, 35015 Republic of Korea; 3 Department of Medical Science, Chungnam National University School of Medicine, 266 Munhwaro, Daejeon, 35015 Republic of Korea; 4 Laboratory of Liver Research, Biomedical Science and Engineering Interdisciplinary program, Korea Advanced Institute of Science and Technology, Daejeon, 34141 Republic of Korea; 5 Department of Internal Medicine, Chungnam National University Hospital, 282 Munhwaro, Daejeon, 35015 Republic of Korea

## Abstract

Growth differentiation factor 15 (GDF15) has recently been shown to have an important role in the regulation of mitochondrial function and in the pathogenesis of complex human diseases. Nevertheless, the role of GDF15 in alcohol-induced or fibrotic liver diseases has yet to be determined. In this study, we demonstrate that alcohol- or carbon tetrachloride (CCl_4_)-mediated hepatic GDF15 production ameliorates liver inflammation and fibrosis. Alcohol directly enhanced GDF15 expression in primary hepatocytes, which led to increased oxygen consumption. Moreover, GDF15 reduced the expression of pro-inflammatory cytokines in liver-resident macrophages, leading to an improvement in inflammation and fibrosis in the liver. GDF15 knockout (KO) mice had more TNF-α-producing T cells and more activated CD4^+^ and CD8^+^ T cells in the liver than wild-type mice. Liver-infiltrating monocytes and neutrophils were also increased in the GDF15 KO mice during liver fibrogenesis. These changes in hepatic immune cells were associated with increased tissue inflammation and fibrosis. Finally, recombinant GDF15 decreased the expression of pro-inflammatory cytokines and fibrotic mediators and prevented the activation of T cells in the livers of mice with CCl_4_-induced liver fibrosis. These results suggest that GDF15 could be a potential therapeutic target for the treatment of alcohol-induced and fibrotic liver diseases.

## Introduction

The liver can be considered an immunologic organ, in which antigen-rich blood from the gastrointestinal tract interacts with diverse innate and adaptive immune cells^1,2^. These immune cells play important roles in the development of hepatic inflammation, steatohepatitis, and fibrotic liver diseases^3,4^. Hepatic macrophages have been implicated in the inflammation induced by hyperglycemia in mice^5^. Natural killer T cells and gamma delta T cells are involved in alcoholic liver injury and hepatic fibrosis, respectively^6,7^. Additionally, migration and activation of neutrophils increase alcohol-induced liver injury^8^. Therefore, many studies have focused on alleviating the secretion of pro-inflammatory cytokines produced by immune cells to treat chronic liver diseases^9,10^.

Growth differentiation factor 15 (GDF15), a member of the transforming growth factor beta superfamily, has anti-inflammatory activities through a currently unknown receptor^11^. In previous reports, serum levels of GDF15 were enhanced in patients with viral hepatitis, cancer, or metabolic disease compared to healthy controls^12–15^. Mitochondrial dysfunction was also associated with elevated serum GDF15 levels in obese mice, which may be a physiologic response to restore metabolic homeostasis^16^. Therefore, GDF15 induction in various inflammatory diseases is thought to be an adaptation to stress response signaling pathways activated by mitochondrial stress.

Hepatocytes display an eosinophilic cytoplasm upon hematoxylin-eosin staining, reflecting abundant mitochondria^17^. Thus, it is not surprising that mitochondrial dysfunction promotes cellular damage and is linked to liver diseases. Chronic alcohol consumption alters mitochondrial oxidative phosphorylation in the liver by suppressing the synthesis of respiratory complex proteins^18^. Alcohol-mediated damage of mitochondrial DNA (mtDNA) also impairs cellular energy metabolism via enhanced formation of reactive oxygen species (ROS)^19^. Additionally, carbon tetrachloride (CCl_4_) reduces mitochondrial respiratory chain complex IV activity and depletes mtDNA in the liver^20^. Moreover, the radicals produced by cytochrome P450 2E1-mediated CCl_4_ metabolism bind to mtDNA directly and also promote lipid peroxidation, which results in degradation of mtDNA^20,21^. Although hepatotoxic molecules such as alcohol and CCl_4_ promote mitochondrial dysfunction in the liver, the role of GDF15 as a mitohormetic factor in alcohol- and CCl_4_-induced liver injury remains to be elucidated.

In this study, we aimed to establish a direct link between mitochondrial function and GDF15 induction. We also explored the anti-inflammatory role of GDF15 in the development of alcohol- and CCl_4_-induced liver injury and examined whether deficiency of GDF15 exacerbates liver injury and fibrosis in mice. Therefore, this study provides a range of pathophysiological insights into alcoholic and fibrotic liver diseases.

## Research Design and Methods

### Mice and ethical considerations

Wild-type (WT) mice on a C57BL/6 background were purchased from the Jackson Laboratory (Bar Harbor, ME, USA). GDF15 KO mice derived from the inbred C57BL/6 strain were provided by Dr. S. Lee (Johns Hopkins University School of Medicine, Baltimore, MD, USA). All mice were maintained in a specific pathogen-free animal facility (Chungnam National University Hospital Preclinical Research Center) in a controlled environment (12 h light/12 h dark cycle; humidity, 50–60%; ambient temperature, 22 ± 2 °C). Mice were placed on a Lieber-DeCarli low-fat liquid diet (Dyets, Dyets, Inc., Bethlehem, PA, USA) containing 1 kcal/ml, of which 18% was derived from protein, 12% from fat, and either 70% from carbohydrate (control diet) or 43% from carbohydrate and 27% from ethanol (alcohol diet). Alcohol was administered gradually by escalating the content by 1% (v/v) each day until the mice were consuming a diet containing 5% (v/v) ethanol. This was continued for six more weeks. At the end of this period, mice were sacrificed, and liver tissues and whole blood were collected for further analysis. All animal experiments were approved by the Institutional Review Board on Animal Experimentation of Chungnam National University School of Medicine (CNUH-A0001) and performed in accordance with the guidelines and regulations of Chungnam National University.

### Carbon tetrachloride (CCl_4_)-induced liver fibrosis in mice

In age-matched (8-week-old) WT and GDF15 KO male mice, liver fibrosis was induced by intraperitoneal injection of CCl_4_ (Sigma-Aldrich, MO; 2 mL/kg in olive oil, 20% v/v) three times per week for 3 weeks. Mice were sacrificed 12 hours after the final CCl_4_ injection.

### Serum biochemical measurements

Serum was collected from whole blood by centrifugation at 10,000 rpm for 5 minutes at room temperature and was assayed for aspartate aminotransferase (AST) and alanine aminotransferase (ALT) using a Fuji Dri-Chem 4000i analyzer (Fujifilm, Tokyo, Japan). Serum or hepatic levels of tumor necrosis factor-alpha (TNF-α) and interleukin-6 (IL-6) were measured using an enzyme linked immunosorbent assay (ELISA) kit (R&D Systems, Minneapolis, MN, USA).

### Histological analyses

Sections of the left and medial lobes of the liver were fixed with 10% neutral buffered formalin (BBC Biochemical, Mt. Vernon, WA, USA) or frozen in Tissue-Tek OCT compound (Sakura, Tokyo, Japan). For formalin-fixed samples, after deparaffinization in xylene and rehydration with ethanol, 4 μm-thick sections were stained with hematoxylin and eosin (H&E) and Sirius Red (Sigma-Aldrich, MO, USA) for detection of collagen deposition in the liver. Frozen sections (10 μm thick) were stained with Oil Red O Kit (ab150678; Abcam, Cambridge, UK) for visualization of lipid accumulation in the liver tissue.

### Measurement of hepatic triglyceride levels

Hepatic lipids were extracted from 100 mg liver tissue using a mixture of chloroform and methanol (2:1 ratio), as previously described^22^. Hepatic lipid extracts were lyophilized with nitrogen gas, and the triglyceride (TG) levels in resuspended lipids were analyzed on a Fuji Dri-Chem 4000i analyzer according to the manufacturer’s instructions (Fujifilm, Tokyo, Japan).

### Isolation of hepatocytes in mice

As previously described^23,24^, mouse livers were perfused *in situ* with EGTA solution (5.4 mM KCl, 0.44 mM KH_2_PO_4_, 140 mM NaCl, 0.34 mM Na_2_HPO_4_, 0.5 mM EGTA, 25 mM Tricine, pH 7.2), followed by the perfusion with a digestion buffer (0.8 mg/ml collagenase type I in HBSS; Worthington, Freehold, NJ, USA) for 30 minutes. The liver cell suspensions were filtered through 70 μm nylon cell strainers (BD Falcon, Millville, NJ, USA), and then centrifuged at 1,000 × g for 5 minutes. The pellet was suspended in 40% Percoll (GE Healthcare, Buckingham, UK) and centrifuged at 1,200 × g for 10 minutes at 4 °C. Isolated cells were cultured in DMEM (Welgene, Daegu, South Korea) supplemented with 10% fetal bovine serum (ThermoFisher Scientific, Waltham, MA, USA) and 1% penicillin/streptomycin (Welgene, Daegu, South Korea) at a density of 2 × 10^5^ cells/well on 6-well plates.

### Isolation of liver mononuclear cells

Liver mononuclear cells (MNCs) were isolated as described previously^5,23^. Briefly, liver tissues were cut into small pieces and incubated with pre-warmed media containing dissociation enzymes (Miltenyi Biotec, Bergisch Gladbach, Germany) for 30 minutes at 37 °C. After enzymatic digestion, the liver cell suspensions were quickly homogenized in C-Tubes using the GentleMACS Dissociator (Miltenyi Biotec) on the m_liver_03 program. After removal of debris, the cells were resuspended in phosphate-buffered saline and centrifuged at 1,000 × g for 5 minutes for the elimination of hepatocytes. The supernatant was removed with mechanical suction and filtered through a 70 μm nylon cell strainer (BD Falcon, Millville, NJ). Hepatic MNCs were isolated by centrifugation at 1,200 × g for 10 minutes at 4 °C and resuspended in RPMI-1640 medium (Welgene, Daegu, South Korea).

### Isolation of hepatic stellate cells and Kupffer cells

As described previously^5,23^, hepatic stellate cells (HSCs) were isolated by *in situ* collagenase perfusion of the liver. After anesthesia by intraperitoneal injection with 100–200 mg/kg ketamine/xylazine cocktails, the liver was perfused *in situ* with collagenase type I through the portal vein. Non-parenchymal cells were separated by centrifugation at 500 rpm and 4 °C for 5 minutes. Then, the HSCs and Kupffer cells were isolated by differential centrifugation on a density gradient of 11.5% and 20% OptiPrep (Sigma-Aldrich, MO, USA) at 3,000 rpm and 4 °C for 17 minutes. HSCs and Kupffer cells were located in the upper and lower layers, respectively. Kupffer cells were positively purified by labeling with anti-mouse F4/80 microbeads (Miltenyi Biotec) and subjected to real-time PCR analysis. HSCs and Kupffer cells were treated with alcohol (50–200 mM), lipopolysaccharide (10 ng/mL; Sigma-Aldrich, MO, USA), or recombinant GDF15 (100 ng/mL; R&D Systems, Minneapolis, MN, USA).

### Co-culture hepatocyte with Kupffer cells or HSCs

Mouse Kupffer cells were cultured in the lower chambers of 12-well plates with lipopolysaccharide (10 ng/mL) overnight. Isolated HSCs were cultured in the lower chambers of 12-well plates with lipopolysaccharide (10 ng/mL) for 4 days. The cells were then rinsed two times with PBS, and WT or GDF15 KO hepatocytes were seeded into the Transwell inserts (Corning Inc., Corning, NY). The two cell lines were co-cultured for 3 hours and the co-cultured Kupffer cells and HSCs were subjected to real-time PCR analysis.

### Fluorescence-activated cell sorting

Isolated liver MNCs were resuspended in Dulbecco’s phosphate-buffered saline (DPBS; Welgene, Daegu, South Korea) containing 0.5% BSA and 0.05% sodium azide, and then labeled with fluorescence-activated cell sorting (FACS) antibodies. For blocking of non-specific binding, cells were pre-incubated with mouse CD16/32 Fc block (eBioscience/ThermoFisher Scientific, Waltham, MA, USA) prior to labeling with FACS antibodies. The cells were also stained with eFluor 780-labeled Fixable Viability Dye (eBioscience/ThermoFisher Scientific, Waltham, MA, USA) to exclude dead cells from the analysis. Lymphocytes (CD4 T, CD8 T, and regulatory T cells), monocytes, and neutrophils were analyzed using anti-CD45, anti-CD4, anti-CD8, anti-CD11b, anti-Ly6C, anti-Ly6G, anti-CD25, and anti-Foxp3 antibodies (eBioscience/ThermoFisher Scientific, Waltham, MA, USA). For intracellular cytokine staining, the cells were re-stimulated with phorbol-myristate acetate/ionomycin for 1 hour in the presence of brefeldin A for 5 hours, and then fixed and permeabilized using the BD Cytofix/Cytoperm kit (BD Pharmingen, San Jose, CA). Stained liver MNCs were read on a FACS Canto II flow cytometer (BD Biosciences, San Jose, CA), and data were analyzed using FlowJo software (FlowJo, LLC, Ashland, OR).

### Cell culture and treatment with alcohol or mitochondrial inhibitors

Mouse primary hepatocytes were prepared from 8–10-week-old male WT and GDF15 KO mice (all on a C57BL/6 background) using *in situ* collagenase perfusion^25^. Isolated hepatocytes were maintained in DMEM (Hyclone, Logan, UT, USA) supplemented with 10% fetal bovine serum and 1% penicillin/streptomycin in a humidified incubator containing 5% CO_2_. Primary murine hepatocytes were treated with alcohol (50–100 mM), recombinant GDF15 (100 ng/mL; R&D Systems, Minneapolis, MN, USA) or the oxidative phosphorylation (OXPHOS) complex inhibitors oligomycin (2 µg/mL; Sigma-Aldrich, MO, USA) and rotenone (1 µM; Sigma-Aldrich, MO, USA) for 24 hours.

### Western blot analysis

Western blot analysis was performed using standard methods with commercially available antibodies. Anti-alpha smooth muscle actin (α-SMA, #ab5694) and anti-cytochrome p450 2E1 (CYP2E1, #ab151544) antibodies were purchased from Abcam (Cambridge, UK). Anti-phospho-NF-kB p65 (#3303), anti-NF-kB p65 (#4764), anti-phospho-p38 (#9215), anti-p38 (#9213) and anti-JNK (#9252) antibodies were purchased from Cell Signaling Technology (Beverley, MA, USA). Anti-phospho-JNK (#700031) and anti-GAPDH (#MA5-15738) antibodies were purchased from ThermoFisher Scientific. Anti-GDF15 (#sc-377195) antibody was purchased from Santa Cruz Biotechnology (Santa Cruz, CA, USA). Immunoreactive bands were visualized using alkaline phosphatase-linked anti-rabbit or anti-mouse secondary antibodies, and images were scanned on an ODYSSEY instrument and Image Studio Software (LI-COR Biosciences; Lincoln, NE, USA).

### Real-time polymerase chain reaction

Total RNA was extracted from cells or tissues using TRIzol reagent (ThermoFisher Scientific, Waltham, MA, USA) according to the manufacturer’s instructions. Complementary DNA (cDNA) was prepared from the same quantity of total RNA using M-MLV reverse transcriptase and oligo-dT primers (Invitrogen/ThermoFisher Scientific, Waltham, MA, USA). The resulting cDNA was amplified on a 7500 Fast Real-Time PCR System (Applied Biosystems, Carlsbad, CA) using 2X SYBR Green mix (Applied Biosystems). All reactions were performed in triplicate with the primers listed in Supplementary Table 1. Relative gene expression was calculated using the ΔΔCT method, with normalization to 18 s expression. Values are expressed as the fold change versus the control group.

### Measurement of the oxygen consumption rate

The mitochondrial oxygen consumption rate (OCR) was measured using a Seahorse XF-24 analyzer (Seahorse Bioscience Inc., North Billerica, MA, USA) in 24-well plates. Hepatocytes were seeded at 2 × 10^4^ cells per well 24 h before the analysis. On the day before the OCR measurement, the sensor cartridge was placed into calibration buffer (Seahorse Bioscience) and incubated in a non-CO_2_ incubator at 37 °C. Hepatocytes were washed and incubated in DMEM without sodium bicarbonate. The medium and mitochondrial OXPHOS inhibitors were adjusted to pH 7.4 on the day of the OCR assay. The basal OCR was measured three times, and three readings were taken after the addition of each mitochondrial OXPHOS inhibitor [oligomycin (2 µg/mL) and rotenone (1 µM)]. The basal and post-oligomycin OCRs were calculated by averaging the last three measurements after maintaining a steady state. Coupled respiration was expressed as the percent decrease from basal respiration. Additionally, carbonyl cyanide *m*-chlorophenyl hydrazone (CCCP; 5 µM) was used to measure maximal mitochondrial respiration of the cells. OCR was automatically calculated and recorded by the sensor cartridge and the Seahorse XF-24 software.

### Statistical analysis

Statistical analyses were performed using Stat Graph Prism 6 (GraphPad, San Diego, CA, USA). Data are reported as the mean ± SEM. All data from animal studies were analyzed by two-way repeated-measures ANOVA followed by Bonferroni’s multiple comparisons, one-way ANOVA followed by Tukey’s post-hoc test, or a two-tailed Student’s *t*-test. *P*-values < 0.05 were considered statistically significant.

## Results

### Alcohol and mitochondrial inhibitors increase hepatic expression of GDF15 *in vitro* and *in vivo*

**Figure 1:**
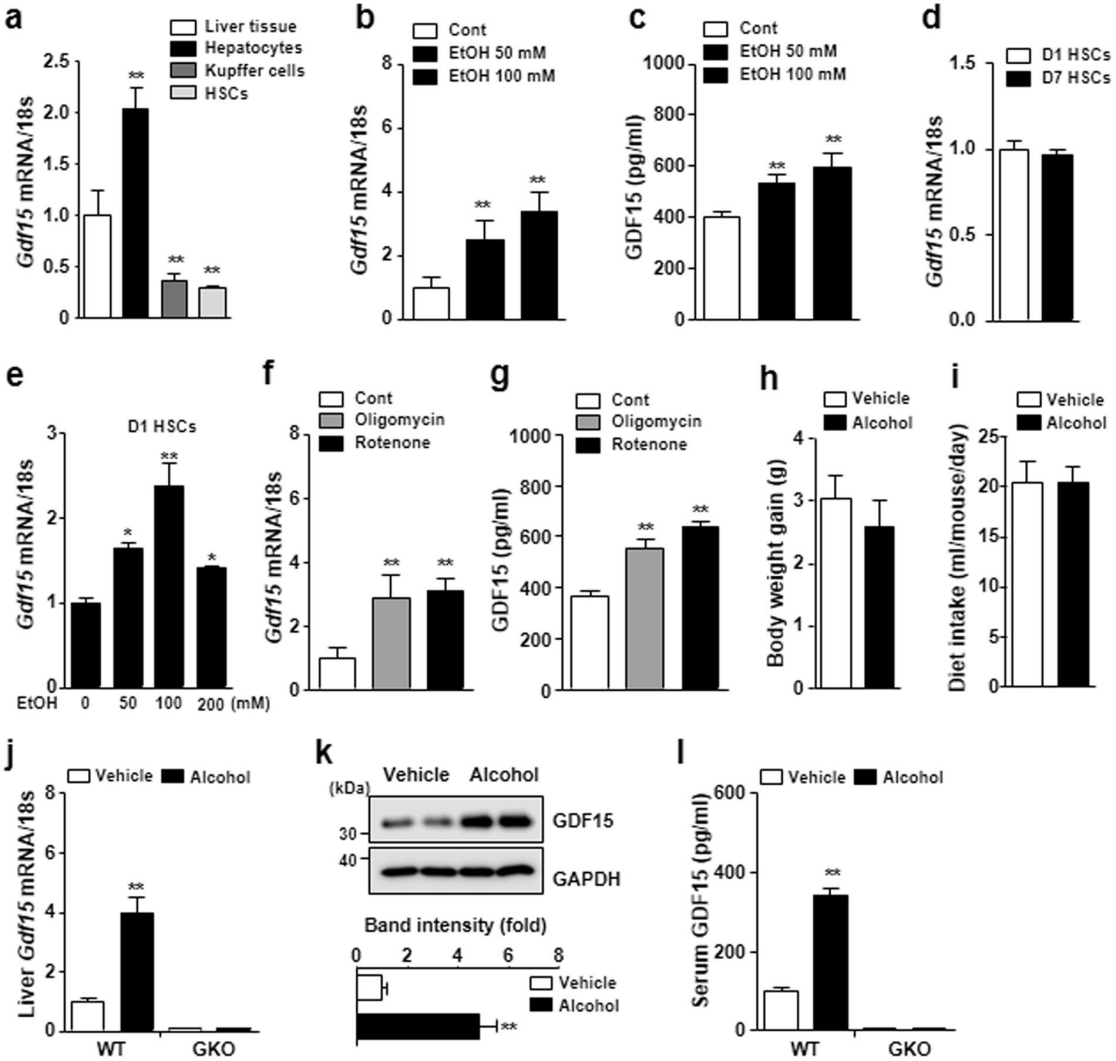
Treatment with alcohol induces GDF15 production in primary murine hepatocytes. (**a**) *Gdf15* expression in liver tissue, hepatocytes, Kupffer cells, and hepatic stellate cells (HSCs). (**b**) Real-time PCR analysis of primary hepatocytes treated with vehicle or alcohol. (**c**) Levels of GDF15 in culture supernatants of primary hepatocytes treated with vehicle or alcohol. (**d**) *Gdf15* expression in D1 HSCs and D7 HSCs (cultured for 1 day and 7 days, respectively). (**e**) *Gdf15* expression in D1 HSCs treated with alcohol. (**f**) Real-time PCR analysis of primary hepatocytes treated with vehicle, oligomycin, or rotenone. (**g**) Levels of GDF15 in culture supernatants of primary hepatocytes treated with vehicle, oligomycin, or rotenone. (**h**,**i**) Body weight gain and dietary intake of mice fed a control or alcohol liquid diet for 6 weeks (n = 5/group). (**j**,**k**) *Gdf15* mRNA and GDF15 protein expression in the livers of WT and GDF15 KO (GKO) mice fed a control or alcohol liquid diet for 6 weeks. Relative quantification of each protein was done by densitometry. (**l**) Serum levels of GDF15 in WT or GKO mice fed vehicle or an alcohol liquid diet for 6 weeks. All data are representative of three independent experiments and are expressed as the mean ± SEM. *P < 0.05 and **P < 0.01, versus the corresponding controls.

Alcohol decreases the activity of the oxidative phosphorylation (OXPHOS) complexes^26^ and increases ROS production^27^, leading to mitochondrial stress or dysfunction. Recently, mitochondrial stress has been shown to promote the induction of GDF15, a mitokine affecting systemic energy metabolism^16^. Based on previous observations that GDF15 expression is associated with the mitochondrial stress response, we examined the effect of alcohol on GDF15 expression in hepatocytes and non-parenchymal cells. As shown in Fig. 1a, *Gdf15* was exclusively expressed in hepatocytes and not HSCs or liver-resident macrophages. *Gdf15* expression was increased in hepatocytes treated with alcohol in a concentration-dependent manner (Fig. 1b). Treatment with alcohol also increased the levels of secreted GDF15 in supernatants of cultured hepatocytes (Fig. 1c). Additionally, we found HSC activation was not related to *Gdf15* expression (Fig. 1d), but that treatment with alcohol increased Gdf15 expression in D1 HSCs (Fig. 1e). We next investigated the effect of mitochondrial dysfunction on GDF15 expression in hepatocytes. Inhibition of the mitochondrial OXPHOS complex using oligomycin or rotenone significantly increased *Gdf15* expression and GDF15 secretion by cultured hepatocytes (Fig. 1f and g). To confirm this observation *in vivo*, WT mice were pair-fed an alcohol or control liquid diet for 6 weeks to investigate the effects of alcohol on GDF15 expression. No significant difference was detected in body weight gain or food intake between the two groups (Fig. 1h and i). Hepatic *Gdf15* expression and serum levels of GDF15 were remarkably increased in alcohol-fed mice (Fig. 1j–l). Taken together, these data demonstrate that treatment with alcohol induces hepatic GDF15 expression *in vitro* and *in vivo*.

### Recombinant GDF15 enhances oxygen consumption by hepatocytes and reduces inflammatory cytokine production by liver-resident macrophages

**Figure 2:**
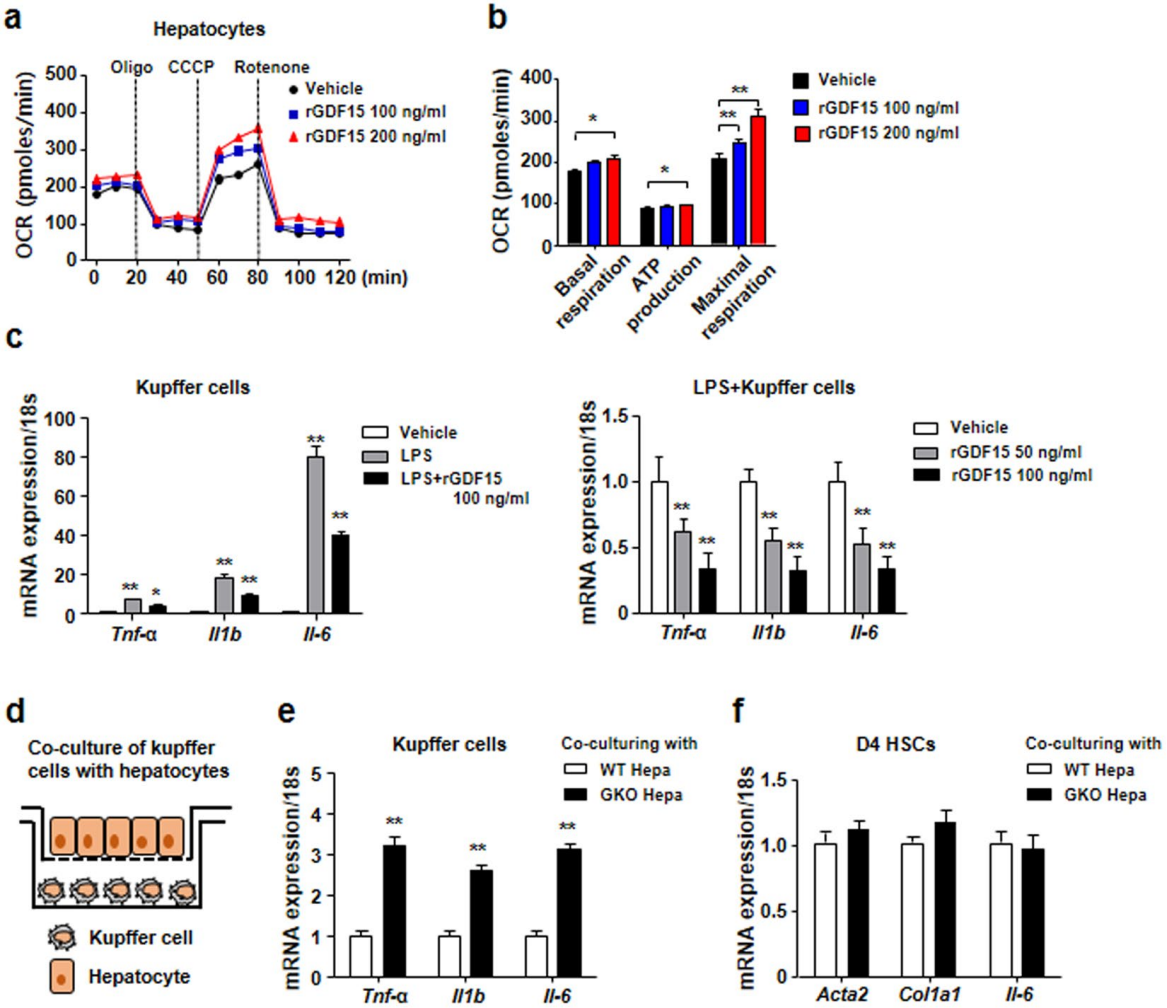
Recombinant GDF15 enhances oxygen consumption in hepatocytes and inhibits the expression of pro-inflammatory cytokines in Kupffer cells. (**a**,**b**) Oxygen consumption rates were measured in primary murine hepatocytes treated with vehicle or recombinant GDF15 (rGDF15, 100 or 200 ng/mL). Oligomycin (2 µg/mL) and rotenone (1 µM) were used as mitochondrial OXPHOS inhibitors. CCCP (5 µM), a mitochondrial uncoupler, was used to measure maximal mitochondrial respiration. (**c**) Real-time PCR analysis of Kupffer cells treated with lipopolysaccharide (LPS; 10 ng/mL) and/or rGDF15 (50 or 100 ng/mL). (**d**) Schematic description of co-culturing Kupffer cells with hepatocytes. (**e**) Real-time PCR analysis of Kupffer cells co-cultured with WT or GDF15 KO hepatocytes in medium containing lipopolysaccharide (10 ng/mL). (**f**) Real-time PCR analysis of HSCs co-cultured with WT or GDF15 KO hepatocytes in medium containing lipopolysaccharide (10 ng/mL) for 3 hours. All data are representative of three independent experiments and are expressed as the mean ± SEM. *P < 0.05 and **P < 0.01, versus the corresponding controls.

Although GDF15 is mainly expressed and secreted by hepatocytes, several types of liver-resident cells, including HSCs, macrophages, and liver sinusoidal endothelial cells as well as hepatocytes can be affected by GDF15. Therefore, hepatocytes were treated with recombinant GDF15 (rGDF15) and the OCR was measured to determine the effect of GDF15 on hepatocyte mitochondrial function. Treatment with rGDF15 increased the basal OCR and the maximal respiration in hepatocytes (Fig. 2a and b). Mitochondrial oxidative metabolism is linked to anti-inflammatory macrophage activation^28^. Therefore, Kupffer cells were treated with lipopolysaccharide (LPS) in the presence or absence of rGDF15 to determine the anti-inflammatory effect of GDF15 on hepatic resident macrophages. GDF15 reduced the expression of pro-inflammatory cytokines in Kupffer cells treated with LPS (Fig. 2c). The anti-inflammatory effect of rGDF15 on LPS-treated Kupffer cells was dose-dependent (Fig. 2c). To further evaluate the paracrine effect of GDF15 released from hepatocytes on Kupffer cells, WT or GDF15 KO hepatocytes were seeded into Transwell inserts, and lipopolysaccharide-treated Kupffer cells were placed in the lower chambers (Fig. 2d). The expression of pro-inflammatory cytokines by the LPS-treated Kupffer cells was significantly increased by co-culture with GDF15 KO hepatocytes as compared to co-culture with WT hepatocytes (Fig. 2e). However, co-culturing intermediate activated D4 HSCs with WT or GDF15 KO hepatocytes in the presence of LPS (10 ng/mL) did not result in any differences in the expression of fibrotic mediators in co-cultured D4 HSCs (Fig. 2f). These data suggest that GDF15 may regulate the hepatic immune microenvironment by inhibiting pro-inflammatory cytokine production from liver-resident macrophages.

### GDF15 deficiency exacerbates alcoholic liver injury in mice

**Figure 3:**
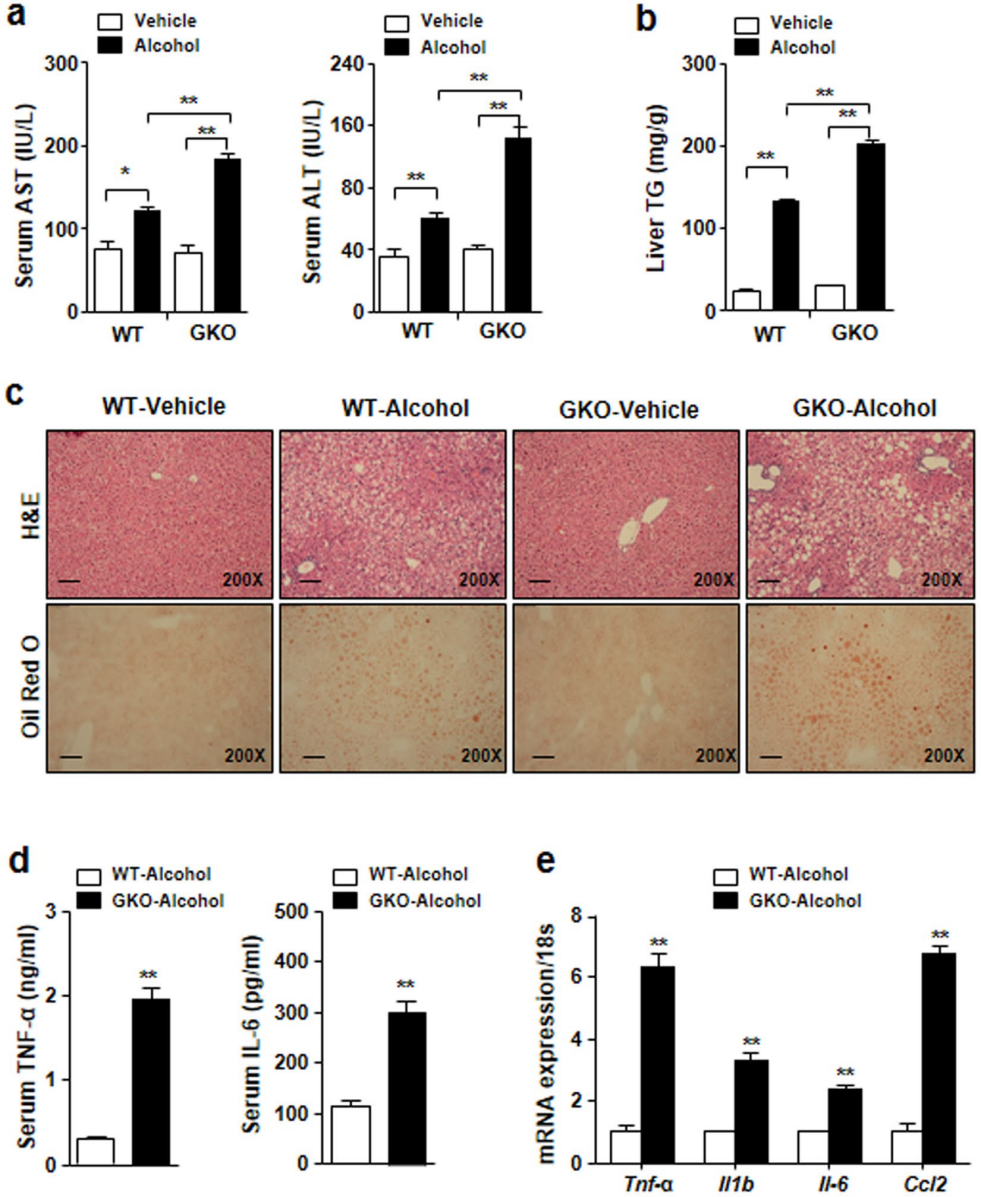
GDF15 protects chronic alcohol-fed mice from liver injury and fat deposition. After 6 weeks of feeding with a control or alcohol liquid diet, WT and GDF15 KO (GKO) mice (n = 6/group) were sacrificed. (**a**) Serum levels of aspartate transaminase (AST) and alanine transaminase (ALT) were measured. (**b**) Triglyceride content in the liver of the mice was measured. (**c**) Hematoxylin-eosin (H&E) and Oil Red O staining of liver sections (original magnification, × 200; bars, 100 μm). (**d**) Serum levels of TNF-α and IL-6. (**e**) Real-time PCR analysis of hepatic *Tnf-α*, *Il1b*, *Il-6*, and *Ccl2*. All data are representative of three independent experiments and are expressed as the mean ± SEM. *P < 0.05 and **P < 0.01, versus the corresponding controls.

To investigate the effect of GDF15 depletion on alcoholic liver injury, WT and GDF15 KO mice were fed an alcohol liquid for 6 weeks. Blood chemistry analysis revealed that serum levels of AST and ALT were increased in alcohol-fed GDF15 KO mice compared to controls (Fig. 3a). Hepatic triglyceride content was also enhanced in the alcohol-fed GDF15 KO mice (Fig. 3b). As shown in Fig. 3c, H&E staining and Oil Red O staining showed that hepatic fat accumulation and immune cell infiltration were increased in alcohol-fed GDF15 KO mice compared to controls (Fig. 3c). Serum levels of TNF-α and IL-6 (Fig. 3d) and hepatic expression of pro-inflammatory cytokines were also increased in alcohol-fed GDF15 KO mice (Fig. 3e). Taken together, these data demonstrate that GDF15 is essential for the regulation of alcohol-mediated liver injury and steatosis.

### GDF15 is required for tissue homeostasis in CCl_4_-induced liver injury and fibrosis

**Figure 4:**
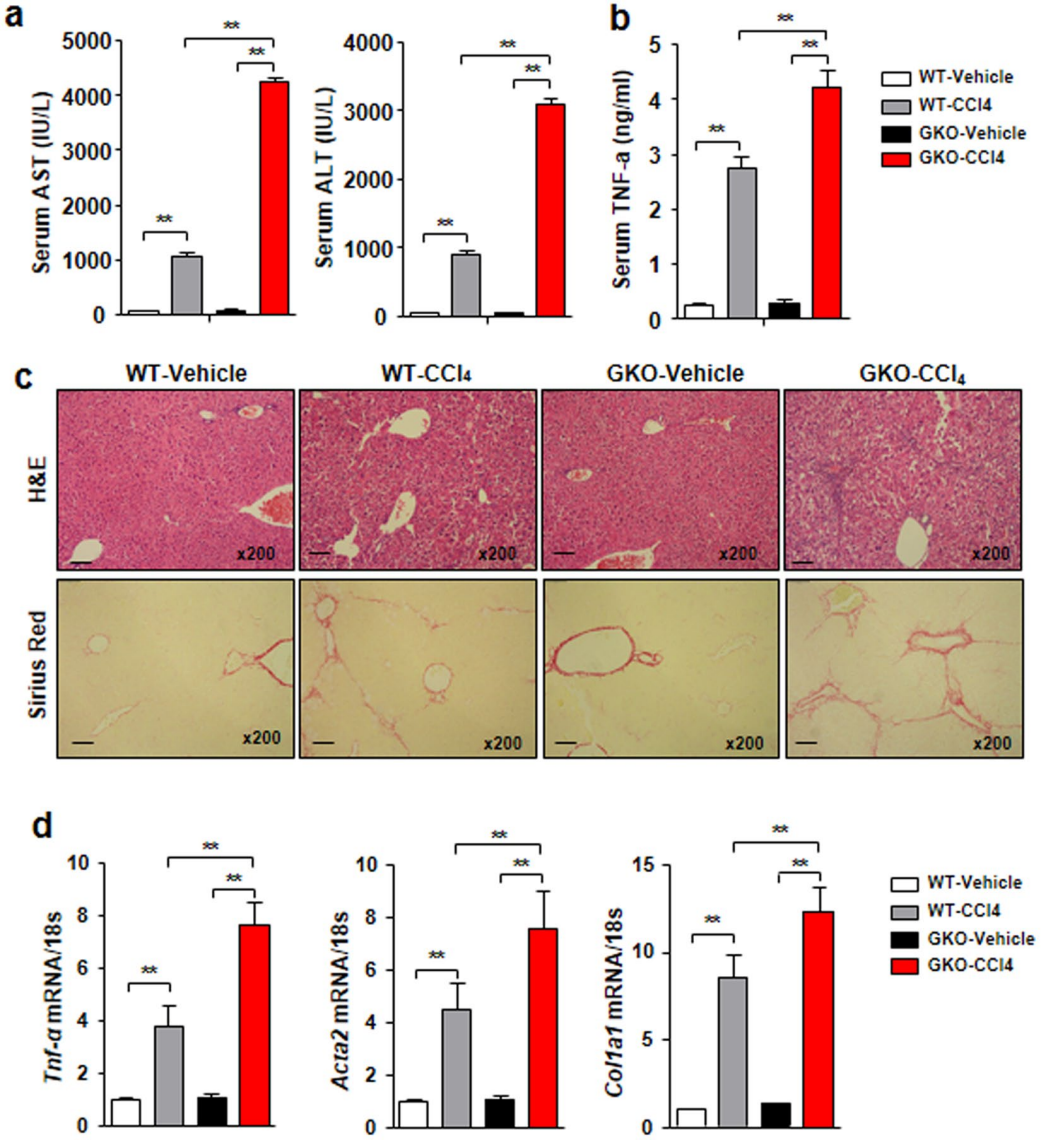
GDF15 deficiency exacerbates CCl_4_-induced liver fibrosis. (**a**) Serum levels of AST and ALT. (**b**) Serum levels of TNF-α. (**c**) H&E and Sirius Red staining of liver sections (original magnification, ×200; bars, 100 μm). (**d**) Real-time PCR analysis of hepatic *Tnf-α*, *Acta2*, and *Col1a1* of the liver from the mice. All data are representative of three independent experiments and are expressed as the mean ± SEM. *P < 0.05, versus the corresponding controls.

Although several animal models for alcoholic liver disease have been developed, they mainly lead to steatosis and low-grade inflammation but not significant fibrosis, even after prolonged administration of alcohol^29–32^. Therefore, to determine the effect of GDF15 on advanced liver disease, we used the CCl_4_-induced model of liver injury. Interestingly, serum levels of AST and ALT were significantly increased in GDF15 KO mice compared to WT mice (Fig. 4a). In addition, serum levels of TNF-α were enhanced in GDF15 KO mice treated with CCl_4_ compared to controls (Fig. 4b). Although there was no significant difference between WT and GDF15 KO mice treated with vehicle, GDF15 KO mice showed more severe liver injury than WT mice treated with CCl_4_ (Fig. 4a and c). In addition, collagen deposition was significantly increased in the livers of GDF15 KO mice treated with CCl_4_ (Fig. 4c). Furthermore, *Tnf-α* and fibrotic mediators such as type 1 collagen 1 alpha 1 (*Col1a1*) and alpha smooth muscle actin (*Acta2*) were upregulated in the livers of GDF15 KO mice treated with CCl_4_ (Fig. 4d). These data demonstrate that GDF15 deficiency induces hepatic inflammation and fibrosis in the CCl_4_-induced model of liver fibrosis.

### GDF15 suppresses activation of hepatic immune cells in CCL_4_-induced liver injury

**Figure 5:**
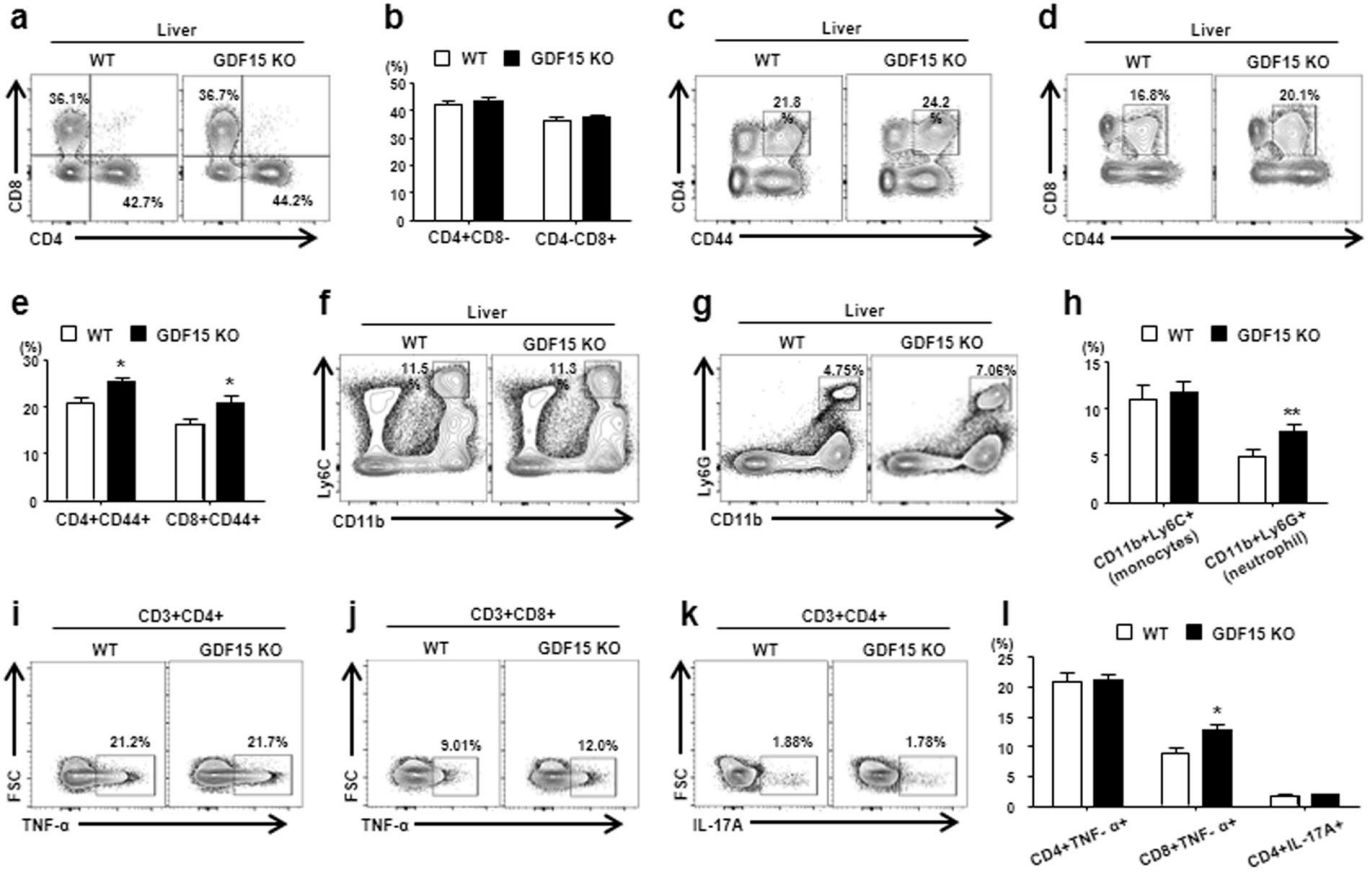
GDF15 suppresses activation of hepatic immune cells during CCl_4_-induced liver fibrogenesis. (**a**,**b**) Percentages of hepatic CD4^+^ and CD8^+^ T cells in WT and GDF15 KO (GKO) mice with liver fibrosis. (**c**–**e**) Percentages of hepatic CD4^+^CD44^+^ and CD8^+^CD44^+^ T cells in WT and GDF15 KO mice with liver fibrosis. (**f**–**h**) Percentages of hepatic monocytes (CD11b^+^Ly6C^high^) and neutrophils (CD11b^+^Ly6G^+^) in WT and GDF15 KO mice with liver fibrosis. (**i**–**l**) Intracellular staining for TNF-α and IL-17A in hepatic CD4^+^ and CD8^+^ T cells. All data are expressed as the mean ± SEM. *P < 0.05 and **P < 0.01, versus the corresponding controls.

To further evaluate the role of GDF15 on hepatic inflammation induced by CCl_4_, the phenotype and function of hepatic immune cells in WT and GDF15 KO mice treated with CCl_4_ were analyzed by FACS. There were no significant differences in the numbers of liver-infiltrating CD4^+^ or CD8^+^ T cells between the two groups (Fig. 5a and b). However, the percentages of activated (CD44^+^) and CD4^+^ and CD8^+^ T cells were significantly increased in the GDF15 KO mice compared to WT mice (Fig. 5c–e). Moreover, the neutrophil (CD11b^+^Ly6G^+^) population was increased in GDF15 KO mice. However, the percentage of monocytes (CD11b^+^Ly6C^high^) in CCl_4_-injured livers was not changed by GDF15 deletion (Fig. 5f–h). Furthermore, the percentage of TNF-α^+^CD8 + T cells in the livers of GDF15 KO mice was higher than in WT mice, but the percentages of TNF-α^+^CD4^+^ T cells and Th17 cells were not significantly different between the two groups (Fig. 5i–l). Collectively, these data indicate that GDF15 is required for the regulation of the hepatic inflammatory response during CCl_4_-mediated liver fibrogenesis.

### Recombinant GDF15 attenuates CCl_4_-induced liver fibrosis by suppressing the hepatic inflammatory response

**Figure 6:**
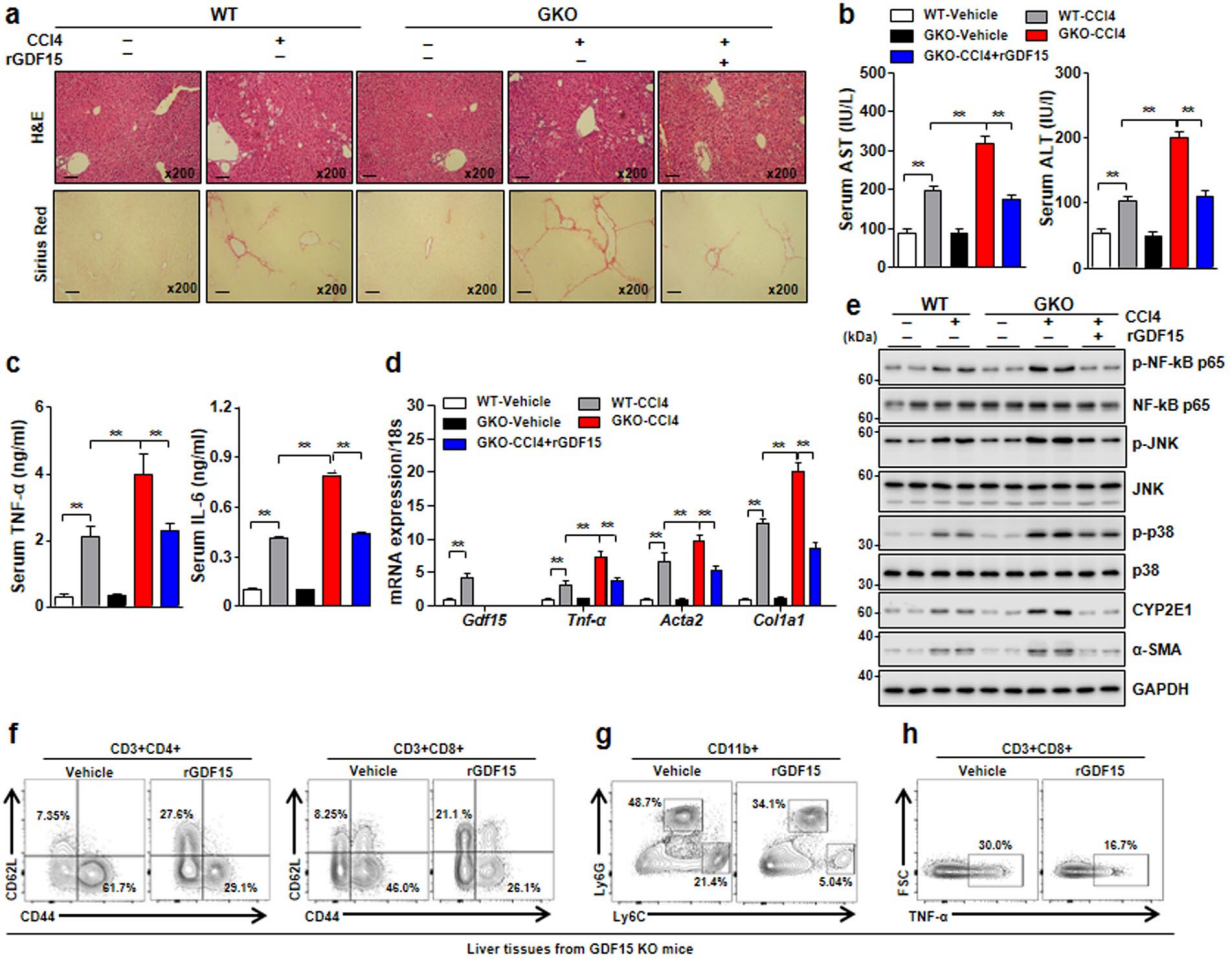
Recombinant GDF15 ameliorates CCl_4_-induced liver inflammation and fibrosis. WT and GDF15 KO (GKO) mice (n = 5/group) were treated with CCl_4_ (2 mL/kg in olive oil, 20% v/v) and recombinant GDF15 (rGDF15, 0.5 mg/kg). (**a**) H&E and Sirius Red staining of liver sections (original magnification, × 200; bars, 100 μm). (**b**) Serum levels of AST and ALT. (**c**) Serum levels of TNF-α and IL-6. (**d**) Real-time PCR analysis of hepatic *Gdf15*, *Tnf-α*, *Acta2*, and *Col1a1*. (**e**) Western blots of liver tissue. (**f**) Percentages of hepatic CD4^+^CD44^+^ and CD8^+^CD44^+^ T cells in GDF15 KO mice treated with vehicle or rGDF15. (**g**) Percentages of hepatic monocytes (CD11b^+^Ly6C^high^) and neutrophils (CD11b^+^Ly6G^+^) in GDF15 KO mice treated with vehicle or rGDF15. (**h**) Intracellular staining for TNF-α in CD8^+^ T cells in the livers of GDF15 KO mice treated with vehicle or rGDF15. All data are expressed as the mean ± SEM. **P < 0.01, versus the corresponding controls.

Based on the data described above, GDF15 deficiency exacerbates liver inflammation and fibrosis. Thus, to investigate the beneficial effect of GDF15 in liver fibrosis, mice were co-injected with CCl_4_ and rGDF15 for 3 weeks. rGDF15 administration reduced collagen accumulation in GDF15 KO mice during liver fibrogenesis (Fig. 6a). As shown in Fig. 6b, serum levels of AST and ALT were lower in GDF15 KO mice after treatment with rGDF15 than GDF15 KO mice treated with CCl_4_ alone. Treatment with rGDF15 also reduced the serum levels of pro-inflammatory cytokines such as TNF-α and IL-6 in the GDF15 KO mice (Fig. 6c). Consistent with these findings, hepatic expression of *Tnf-α*, *Acta2*, and *Col1a1* was significantly downregulated in GDF15 KO by treatment with rGDF15 (Fig. 6d). To decipher the molecular basis by which rGDF15 reduces CCl_4_-induced liver injury and fibrosis, phosphorylation of NF-kB, JNK, and p38 was evaluated in the livers of WT and GDF15 KO mice treated with CCl_4_ and rGDF15. NF-kB phosphorylation was significantly increased by injection of CCl_4_ in GDF15 KO mice, while treatment with rGDF15 reduced CCl_4_-induced phosphorylation of NF-kB and JNK in the livers of GDF15 KO mice (Fig. 6e). CCl_4_-induced p38 phosphorylation was also significantly decreased in the livers of GDF15 KO mice treated with rGDF15 (Fig. 6e). Additionally, CYP2E1 expression was downregulated in the livers of GDF15 KO mice treated with rGDF15 (Fig. 6e). This pharmacologic inhibition of NF-kB, JNK and CYP2E1 expression and p38 phosphorylation may contribute to the suppression of α-SMA expression in the liver (Fig. 6e), leading to attenuation of CCl_4_-induced liver fibrosis. To analyze the effect of rGDF15 on hepatic resident immune cells, immune infiltrates in the livers of mice with CCl_4_-induced liver injury treated with rGDF15 were analyzed by FACS. Treatment with rGDF15 reduced the percentage of CD4^+^ and CD8^+^ CD44^+^ T cells and increased the percentage of CD4^+^ and CD8^+^ CD62L^+^ T cells in the liver (Fig. 6f). The numbers of monocytes (CD11b^+^Ly6C^high^) and neutrophils in the liver were also significantly decreased in the GDF15 KO mice treated with rGDF15 compared to GDF15 KO mice treated with vehicle (Fig. 6g). Moreover, treatment with rGDF15 reduced the numbers of CD8^+^TNF-α^+^ T cells in the livers of GDF15 KO mice (Fig. 6h). These data demonstrate that rGDF15 regulates hepatic immune homeostasis, thereby ameliorating CCl_4_-induced liver injury and fibrosis.

## Discussion

**Figure 7:**
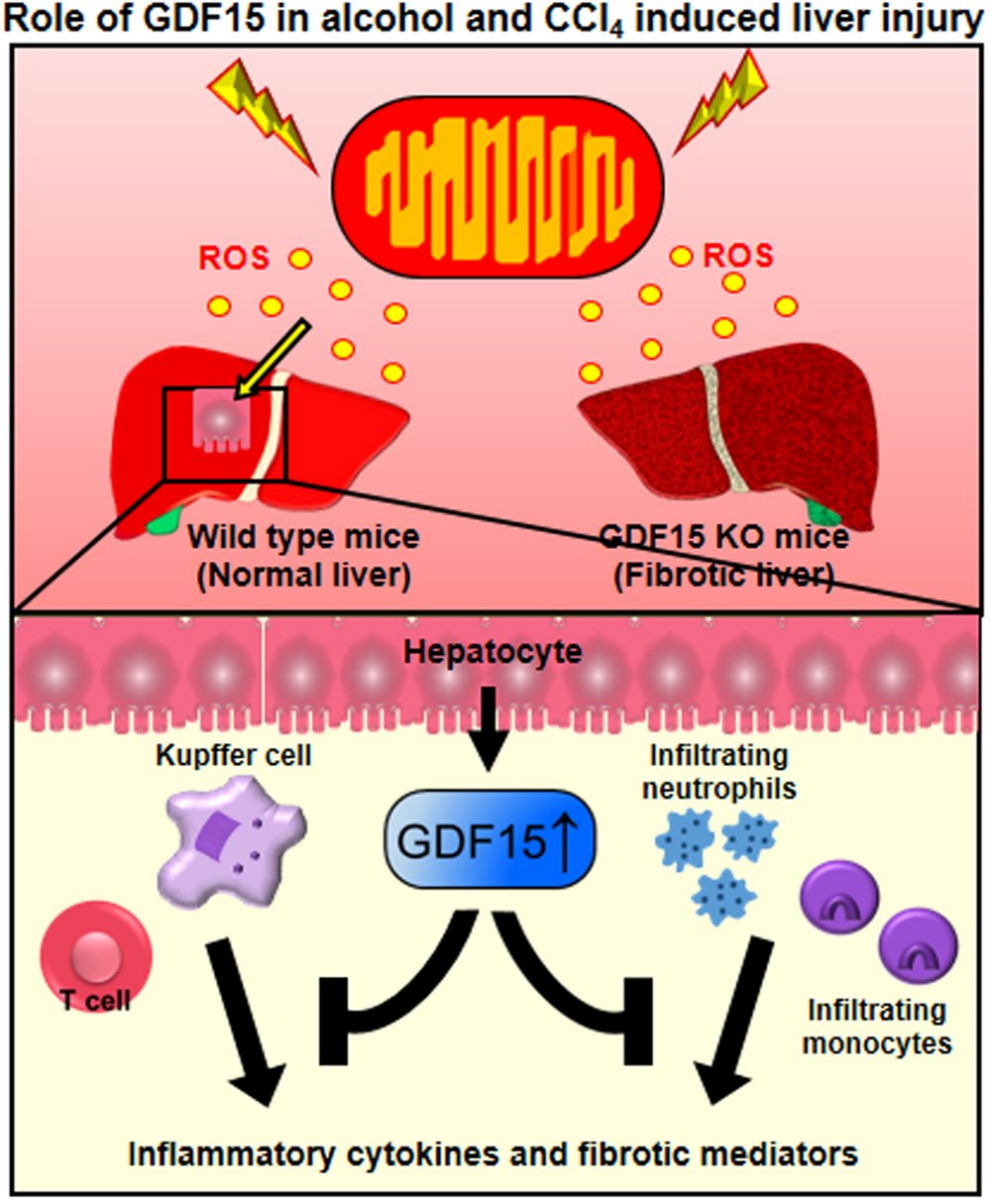
Graphical summary. Alcohol and CCl_4_ increase GDF15 production in hepatocytes. During alcohol-induced and fibrotic liver injury, GDF15 decreases hepatic inflammation and fibrosis via regulation of liver immune cells.

The link between serum levels of GDF15 and liver disease is well described in mice and in humans, but it remains to be determined whether GDF15 has direct effects on hepatic inflammation and fibrosis. In the present study, we clearly show that alcohol and CCl_4_ promote hepatic GDF15 induction, which is required for protection from alcohol- and CCl_4_-induced liver injury in mice. The findings of this study are summarized in Fig. 7.

Although the GDF15 receptor and the signaling pathways that drive its biological action have not been unequivocally elucidated, GDF15 is currently considered a reliable biomarker for liver disease, cardiovascular disease, and cancer^12–14,33^. Moreover, GDF15 is induced by the cellular adaptive response to tissue injury or exposure to carcinogens in a number of solid organs^34,35^. However, most data on the relationship between GDF15 and disease come from observational studies, and there is no conclusive evidence on causality. In the current study, we show that alcohol and CCl_4_-induced liver inflammation and fibrosis are more severe in GDF15 KO mice, which suggests a causal relationship between GDF15 and liver disease. These data demonstrate that GDF15 is a negative regulator of hepatic inflammation and fibrosis.

Recent emerging evidence has suggested that cellular GDF15 is involved in mitochondrial dysfunction in humans and rodents. Specifically, serum levels of GDF15 are increased in patients with mitochondrial disease^36^. Moreover, mitochondrial OXPHOS dysfunction in skeletal muscle promotes GDF15 expression in mice^16^. Meanwhile, mitochondria are abundant in liver cells and are essential in hepatic metabolism, cell survival, and ROS homeostasis^37^. Specifically, mitochondria are more involved in glucose, lipid, and protein metabolism in the liver than in other organs^37^. Therefore, hepatic mitochondrial dysfunction is implicated in the pathogenesis of liver diseases such as alcoholic and non-alcoholic steatohepatitis, insulin resistance, and fibrosis. Chronic alcohol feeding alters the expression of respiratory chain complexes in the liver mitochondria^38^, and patients with non-alcoholic steatohepatitis show morphological changes in the liver mitochondria^39^. Heterozygosity for the mitochondrial trifunctional protein promotes hepatic fat accumulation and insulin resistance in mice^40^. Moreover, mitochondrial dysfunction in hepatocytes has a critical role in the progression of CCl_4_-induced liver fibrosis in mice^41^. However, recently, mild mitochondrial perturbation in the muscle increased the lifespan and delayed age-dependent locomotory impairment in *Drosophila* ^42^. The protective effects of muscle mitohormesis may be associated with the mitochondrial unfolded protein response, which is caused by dysfunction of the mitochondrial respiratory chain complex I^42^. Additionally, reduced mitochondrial OXPHOS function in muscle improved systemic energy homeostasis and glucose intolerance via GDF15 secretion from muscle in mice^16^. In the present study, we also showed that treatment with mitochondrial inhibitors such as oligomycin and rotenone induced GDF15 production in primary murine hepatocytes (Fig. 1), which may drive the protective effect against alcohol- or CCl_4_-induced liver injury. This evidence suggests that mitochondrial dysfunction may be a double-edged sword, as it may affect disease progression as well as protection.

The liver can be regarded as an immunologic organ, with diverse resident and infiltrating immune cells^2^. These immune cells contribute to the maintenance of hepatic immune homeostasis, but dysregulation of immune cells is involved in the development of inflammation and fibrosis in the liver. We have investigated the effect of GDF15 on the number and function of hepatic immune cells in WT and GDF15 KO mice with CCl_4_-induced liver injury. As shown in Fig. 5, the numbers of CD4^+^CD44^+^ T cells, CD8^+^CD44^+^ T cells, and TNF-α-producing CD8^+^ T cells were increased in GDF15 KO mice compared to WT mice during CCl_4_-induced liver fibrogenesis. Additionally, treatment with rGDF15 reduced the numbers of CD8^+^TNF-α^+^ T cells and the numbers of activated CD4^+^ and CD8^+^ T cells, but increased the numbers of naïve CD4^+^ and CD8^+^ T cells in the livers of GDF15 KO mice (Fig. 6). These findings suggest that GDF15 has an anti-inflammatory role in CCl_4_-induced liver injury, resulting in reduced collagen deposition in the liver. In agreement with our results, a recent study also showed that GDF15 inhibited the inflammatory response and septic heart and renal injury induced by lipopolysaccharide^43^. Furthermore, NF-kB, JNK, and p38 signaling pathways, which are closely associated with hepatic inflammation and fibrosis^44^, were inhibited by treatment with rGDF15 in a mouse model of liver fibrosis (Fig. 6). This result is also consistent with the anti-inflammatory effect of GDF15 in hepatic immune cells. However, further evaluation is required to clarify whether GDF15 directly reduces the numbers and functional activity of immune cells in the liver, and to identify the molecular mechanism responsible for the GDF15 effect on signaling pathways.

In a previous investigation, GDF15 did not affect the regeneration capacity of the liver after acute liver injury with a single injection of CCl_4_^45^. Liver regeneration, as assessed by BrdU labeling, was also not different between WT and GDF15 KO mice after partial hepatectomy and a single CCl_4_ injection^45^. By contrast, our study showed that GDF15 has anti-inflammatory and anti-fibrotic roles in CCl_4_-induced chronic (3 week) liver fibrosis, which is more generally used to induce liver fibrosis than a single CCl_4_ injection. Additionally, we suggested that liver fibrosis can be attenuated by the anti-inflammatory action of GDF15, based on the observation that the numbers of neutrophils and activated T cells were lower in the inflamed liver of mice treated with rGDF15. However, the strain (C57BL/6) of GDF15 KO mice used in our study was different from the mouse strain (129/SvJ) used in their study, indicating that the differences between these two studies could be due to differences in experimental methodology and the genetic background of the mice.

Many previous investigations have revealed a remarkable induction of GDF15 in a number of diseases, such as inflammation, cancer, and various metabolic diseases, suggesting that it is induced as a compensatory measure. Alcohol- and CCl4-induced liver injury also induces a remarkable increase in GDF15 levels in WT mice. Because the existing high levels of GDF15 in various injury models make it difficult to pinpoint the role of rGDF15 in WT mice, we administered rGDF15 to GDF15 KO mice to obtain a clearer picture of the role played by GDF15 after hepatic cell injury. However, to determine the therapeutic role of GDF15, rGDF15 should be administered to WT mice rather than to GDF15 KO mice. Therefore, further study is required to establish a therapeutic role for alcoholic steatosis and fibrotic liver diseases.

In summary, we demonstrate that GDF15 has an anti-inflammatory role in alcohol- and CCl_4_-induced liver injury in mice. Mitochondrial dysfunction caused by alcohol and CCl_4_ promotes GDF15 production in hepatocytes, which inhibits alcohol-mediated fat accumulation and CCl_4_-induced fibrosis in the liver. Although future studies should be performed to elucidate the mechanism of cellular GDF15 induction and secretion in damaged hepatocytes, GDF15 could be a novel therapeutic target for the treatment of liver diseases.

## Electronic supplementary material

Supplementary information

## References

[1] Racanelli V, Rehermann B (2006). The liver as an immunological organ. Hepatology.

[2] Yi HS, Jeong WI (2013). Interaction of hepatic stellate cells with diverse types of immune cells: foe or friend. Journal of gastroenterology and hepatology.

[3] Tacke F, Luedde T, Trautwein C (2009). Inflammatory pathways in liver homeostasis and liver injury. Clinical reviews in allergy & immunology.

[4] Tsuchida, T. & Friedman, S. L. Mechanisms of hepatic stellate cell activation. Nature reviews. *Gastroenterology & hepatology*, doi:10.1038/nrgastro.2017.38 (2017). 10.1038/nrgastro.2017.38 28487545

[5] Lee YS (2016). Hepatic immunophenotyping for streptozotocin-induced hyperglycemia in mice. Scientific reports.

[6] Cui K (2015). Invariant NKT cells promote alcohol-induced steatohepatitis through interleukin-1beta in mice. Journal of hepatology.

[7] Seo W (2016). Exosome-mediated activation of toll-like receptor 3 in stellate cells stimulates interleukin-17 production by gammadelta T cells in liver fibrosis. Hepatology.

[8] Kono H (2001). ICAM-1 is involved in the mechanism of alcohol-induced liver injury: studies with knockout mice. American journal of physiology. Gastrointestinal and liver physiology.

[9] Hill DB (2002). A role for interleukin-10 in alcohol-induced liver sensitization to bacterial lipopolysaccharide. Alcoholism, clinical and experimental research.

[10] Louis H (1998). Interleukin-10 controls neutrophilic infiltration, hepatocyte proliferation, and liver fibrosis induced by carbon tetrachloride in mice. Hepatology.

[11] Breit SN (2011). The TGF-beta superfamily cytokine, MIC-1/GDF15: a pleotrophic cytokine with roles in inflammation, cancer and metabolism. Growth factors.

[12] Hong JH (2014). *GDF15 Is a Novel Biomarker for I*mpaired Fasting Glucose. Diabetes & metabolism journal.

[13] Liu X (2015). Association of serum level of growth differentiation factor 15 with liver cirrhosis and hepatocellular carcinoma. PloS one.

[14] Mehta RS (2014). A prospective study of macrophage inhibitory cytokine-1 (MIC-1/GDF15) and risk of colorectal cancer. Journal of the National Cancer Institute.

[15] Wang X (2014). Macrophage inhibitory cytokine 1 (MIC-1/GDF15) as a novel diagnostic serum biomarker in pancreatic ductal adenocarcinoma. BMC cancer.

[16] Chung HK (2017). Growth differentiation factor 15 is a myomitokine governing systemic energy homeostasis. The Journal of cell biology.

[17] Nassir F, Ibdah JA (2014). Role of mitochondria in alcoholic liver disease. World journal of gastroenterology.

[18] Wei YH, Chen YS, Lee JF, Huang JY, Lee CH (1990). Effect of ethanol intake on rat liver mitochondrial respiration and oxidative phosphorylation. Proceedings of the National Science Council, Republic of China. Part B, Life sciences.

[19] Mansouri A (1999). An alcoholic binge causes massive degradation of hepatic mitochondrial DNA in mice. Gastroenterology.

[20] Knockaert L (2012). Carbon tetrachloride-mediated lipid peroxidation induces early mitochondrial alterations in mouse liver. Laboratory investigation; a journal of technical methods and pathology.

[21] Levy GN, Brabec MJ (1984). Binding of carbon tetrachloride metabolites to rat hepatic mitochondrial DNA. Toxicology letters.

[22] Folch J, Lees M, Sloane Stanley GH (1957). A simple method for the isolation and purification of total lipides from animal tissues. The Journal of biological chemistry.

[23] Yi HS (2014). Alcohol dehydrogenase III exacerbates liver fibrosis by enhancing stellate cell activation and suppressing natural killer cells in mice. Hepatology.

[24] Kang SG (2017). ANGPTL6 expression is coupled with mitochondrial OXPHOS function to regulate adipose FGF21. The Journal of endocrinology.

[25] Chung HK (2015). The indole derivative NecroX-7 improves nonalcoholic steatohepatitis in ob/ob mice through suppression of mitochondrial ROS/RNS and inflammation. Liver Int.

[26] Bailey SM, Cunningham CC (1999). Effect of dietary fat on chronic ethanol-induced oxidative stress in hepatocytes. Alcoholism, clinical and experimental research.

[27] Robin MA (2005). Ethanol increases mitochondrial cytochrome P450 2E1 in mouse liver and rat hepatocytes. FEBS letters.

[28] Vats D (2006). Oxidative metabolism and PGC-1beta attenuate macrophage-mediated inflammation. Cell metabolism.

[29] Bertola A, Mathews S, Ki SH, Wang H, Gao B (2013). Mouse model of chronic and binge ethanol feeding (the NIAAA model). Nature protocols.

[30] Brandon-Warner E, Schrum LW, Schmidt CM, McKillop IH (2012). Rodent models of alcoholic liver disease: of mice and men. Alcohol.

[31] Leo MA, Lieber CS (1983). Hepatic fibrosis after long-term administration of ethanol and moderate vitamin A supplementation in the rat. Hepatology.

[32] Mathews S, Xu M, Wang H, Bertola A, Gao B (2014). Animals models of gastrointestinal and liver diseases. Animal models of alcohol-induced liver disease: pathophysiology, translational relevance, and challenges. American journal of physiology. Gastrointestinal and liver physiology.

[33] Wallentin L (2013). GDF-15 for prognostication of cardiovascular and cancer morbidity and mortality in men. PloS one.

[34] Zimmers TA (2005). Growth differentiation factor-15/macrophage inhibitory cytokine-1 induction after kidney and lung injury. Shock.

[35] Zimmers TA (2006). Growth differentiation factor-15: induction in liver injury through p53 and tumor necrosis factor-independent mechanisms. The Journal of surgical research.

[36] Montero R (2016). GDF-15 Is Elevated in Children with Mitochondrial Diseases and Is Induced by Mitochondrial Dysfunction. PloS one.

[37] Degli Esposti D (2012). Mitochondrial roles and cytoprotection in chronic liver injury. Biochemistry research international.

[38] Han D (2012). Dynamic adaptation of liver mitochondria to chronic alcohol feeding in mice: biogenesis, remodeling, and functional alterations. The Journal of biological chemistry.

[39] Caldwell SH (1999). Mitochondrial abnormalities in non-alcoholic steatohepatitis. Journal of hepatology.

[40] Ibdah JA (2005). Mice heterozygous for a defect in mitochondrial trifunctional protein develop hepatic steatosis and insulin resistance. Gastroenterology.

[41] Mitchell C (2009). Protection against hepatocyte mitochondrial dysfunction delays fibrosis progression in mice. The American journal of pathology.

[42] Owusu-Ansah E, Song W, Perrimon N (2013). Muscle mitohormesis promotes longevity via systemic repression of insulin signaling. Cell.

[43] Abulizi P (2017). Growth Differentiation Factor-15 Deficiency Augments Inflammatory Response and Exacerbates Septic Heart and Renal Injury Induced by Lipopolysaccharide. Scientific reports.

[44] Luedde T, Schwabe RF (2011). NF-kappaB in the liver–linking injury, fibrosis and hepatocellular carcinoma. Nature reviews. Gastroenterology & hepatology.

[45] Hisao, E. C. *et al*. Characterization of growth-differentiation factor 15, a transforming growth factor beta superfamily member induced following liver injury. *Molecular and Cellular Biology* **20**, 3742-3751, doi:10.1128/MCB.20.10.3742-3751.2000. 10.1128/mcb.20.10.3742-3751.2000 PMC85678 10779363

