# Supplementary material for: GDF15 deficiency exacerbates chronic alcohol- and carbon tetrachloride-induced liver injury
Source: Sci Rep. 2017 Dec 8;7:17238. doi: 10.1038/s41598-017-17574-w (PMC5722931; doi:10.1038/s41598-017-17574-w)

**Supplementary Information**

**GDF15 deficiency exacerbates chronic alcohol- and carbon tetrachloride-induced liver injury**

- Hyo Kyun Chung^1,2#^, Jung Tae Kim^1,3,#^, Hyeon-Woo Kim^1,3^, Minjoo Kwon^1^, So Yeon Kim^4^, Minho Shong^1,5^, Koon Soon Kim^1,3*^, Hyon-Seung Yi^1,5*^
- ^1^Research Center for Endocrine and Metabolic Diseases, Chungnam National University School of Medicine, 282 Munhwaro, Daejeon 35015, Republic of Korea
- ^2^Research Institute for Medical Sciences, Chungnam National University School of Medicine, 266 Munhwaro, Daejeon 35015, Republic of Korea

^3^Department of Medical Science, Chungnam National University School of Medicine, 266 Munhwaro, Daejeon 35015, Republic of Korea

- ^4^Laboratory of Liver Research, Biomedical Science and Engineering Interdisciplinary program, Korea Advanced Institute of Science and Technology, Daejeon 34141, Republic of Korea
- ^5^Department of Internal Medicine, Chungnam National University Hospital, 282 Munhwaro, Daejeon 35015, Republic of Korea
- ^#^ These authors contributed equally to this work.

*Correspondence to:

KSK ([kunsunkim@cnu.ac.kr](mailto:kunsunkim@cnu.ac.kr)); [Tel: 82-42-280-7148](tel:82-42-280-7148); Fax: 82-42-280-7995

HSY (jmpbooks@cnuh.co.kr); Tel: 82-42-280-6994; Fax: 82-42-280-7995

**Supplementary Table 1. Primers used for real-time PCR (Mouse)**

| **Gene** | **Forward** | **Reverse** |
| --- | --- | --- |
| *18s* | CTGGTTGATCCTGCCAGTAG | CGACCAAAGGAACCATAACT |
| *Gdf15* | GAGCTACGGGGTCGCTTC | GGGACCCCAATCTCACCT |
| *Tnf-a* | CCCCAAAGGGATGAGAAGTT | CACTTGGTGGTTTGCTACGA |
| *Il1b* | GACCTTCCAGGATGAGGACA | TGTTCATCTCGGAGCCTGTA |
| *Il-6* | CCGGAGAGGAGACTTCACAG | CAGAATTGCCATTGCACAAC |
| *Ccl2* | CCCAATGAGTAGGCTGGAGA | TCTGGACCCATTCCTTCTTG |
| *Acta2* | CTGACAGAGGCACCACTGAA | GAAGGAATAGCCACGCTCAG |
| *Col1a1* | TCCTCCAGGGATCCAACGA | GGCAGGCGGGAGGTCTT |

Supplementary Figure 1. Original images of representative uncropped immunoblots from Figure 1k.


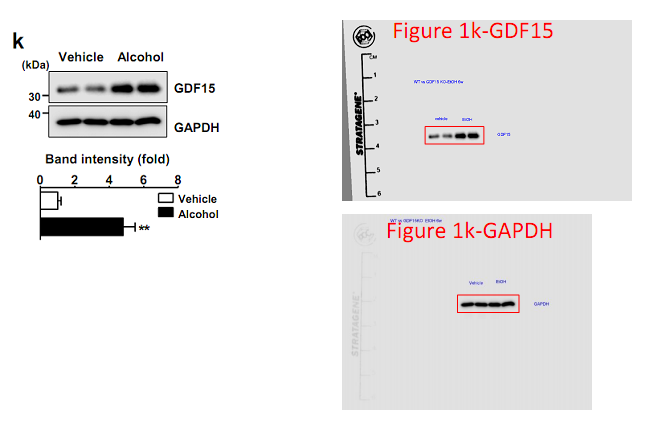


Supplementary Figure 2. Original images of representative uncropped immunoblots from Figure 6e.


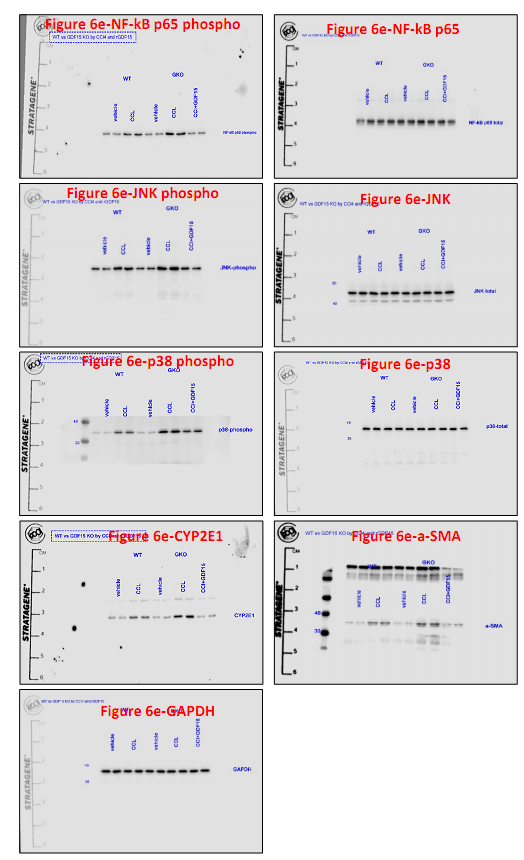

Supplement: Supplementary file 1 — Supplementary information [file 41598_2017_17574_MOESM1_ESM.doc]
